# Zero-Risk Interpretation in the Level of Preventive Action Method Implementation for Health and Safety in Construction Sites

**DOI:** 10.3390/ijerph18073534

**Published:** 2021-03-29

**Authors:** Antonio José Carpio-de los Pinos, María de las Nieves González-García, Ligia Cristina Pentelhão, J. Santos Baptista

**Affiliations:** 1 Department of Applied Mechanics and Project Engineering, School of Industrial and Aerospace Engineering of Toledo, University of Castilla La Mancha, 45071 Toledo, Spain; AntonioJose.Carpio@uclm.es; 2 Departamento Construcciones Arquitectónicas y su Control, Escuela Técnica Superior de Edificación, Universidad Politécnica de Madrid, 28040 Madrid, Spain; 3 Associated Laboratory for Energy, Transports and Aeronautics, LAETA (PROA), Faculty of Engineering, University of Porto, 4200-465 Porto, Portugal; up201902808@fe.up.pt (L.C.P.); jsbap@fe.up.pt (J.S.B.)

**Keywords:** risk assessment, construction work, health and safety, zero-risk

## Abstract

Risk assessment is a legal obligation for all companies in most countries worldwide. It aims to control the quality of working conditions and avoid externalizing the consequences of accidents and resulting costs to society. This work discusses the need for an adequate interpretation of the zero-risk concept from a technical-preventive perspective to assess occupational risks in construction sites. A critical analysis of several risk assessment methodologies was carried out, focusing on the evaluation criteria of little or no-risk situations. The verification of the results was made through a case study. The perception of health and safety risks by workers is very different from that of the evaluators. Often, when workers identify a situation as low-risk or even zero-risk, the evaluator assesses the same context as maximum risk. Given the workers’ and the evaluators’ responses, the Preventive Action Method establishes a new parameter, the Environment Congruence. This parameter is based on the perception of the preventive environment and gives more importance to the evaluators’ decision. When preventive action is optimal, the risk is low in all preventive observation settings. In conclusion, this study justifies the non-nullity of the risk and the difficulty of assessing zero-risk in construction sites. Therefore, evaluations with qualitative and quantitative non-risk approaches should be discarded.

## 1. Introduction

All companies must carry out mandatory risk assessments regarding their workers’ health and safety, regardless of their production and size [1,2]. For this process, several risk assessment methodologies are available to analyze risk from different intervention areas, such as Occupational Safety, Industrial Hygiene, Ergonomics, and Psychosociology [3]; and its implementation can be individually or globally [4]. There are currently many risk assessment methodologies that carry out risk assessment from specific approaches, specifying a discipline to combat risks or a particular risk. However, more recent research exposes the need for risk assessment to be carried out in an integrated way, covering the four main intervention areas (Occupational Safety, Industrial Hygiene, Ergonomics, and Psychosociology) [5], and thereby increasing the effectiveness of risk assessment.

Occupational risk assessment methodologies have two primary application techniques to identify risks. On the one hand, medical procedures act on the individual with periodic medical examinations, health care, and physical rehabilitation [6,7,8]; on the other, different approaches focus on the work environment. The latter includes accident prevention through the diagnosis and prevention of occupational accidents and diseases, the prevention of physical and mental fatigue using ergonomic principles, and the organization of conditions and human relationships using psychosociology [9,10].

**Figure 1:**
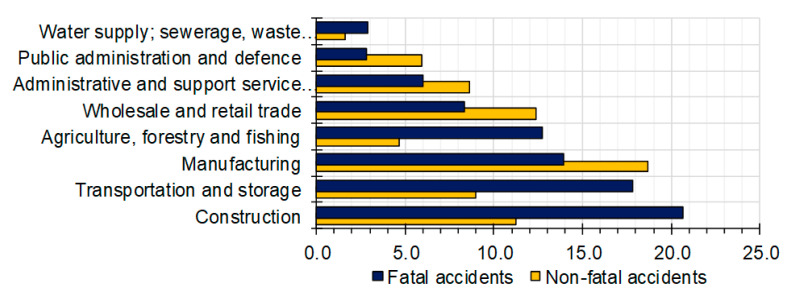
Fatal and non-fatal accidents at work by NACE section, EU-28, 2017 (% of fatal and non-fatal accidents).

The incidence rate for occupational accidents forms an important context for the implementation of occupational risk assessment methodologies [11]. Construction is the sector with the highest number of occupational accidents, which is more than double the average of the other sectors [12,13,14,15]. In 2017, one-fifth of all fatal accidents at work in EU-28 countries occurred within the construction sector (Figure 1) [16].

At construction sites, work is carried out using multiple construction processes associated with specific risks that can cause severe and fatal accidents. Among the constructive systems most common and notable are excavation, earthmoving, construction, assembly and disassembly of prefabricated elements, conditioning of facilities, transformation, rehabilitation, repair, dismantling, demolition, maintenance, conservation, painting, cleaning or sewer work, meetings on-site, weather, and haste for delivery by a specific date [17,18]. One of the particular characteristics of construction sites is that different operations and tasks can occur simultaneously in time and space. This causes the associated risks to overlap, increasing the likelihood of accidents and their consequences [5,10].

Industrial accidents are a major problem both economically and socially and represent a permanent economic loss for businesses, governments, workers, and society in general [19]. Reducing workplace accidents is an important social priority, and accident prevention and risk management are crucial issues within the construction industry [20]. Consequently, learning to observe people in their jobs is essential to identifying unsafe or problematic actions [21,22].

Assessing occupational risks at construction sites is evolving [23]. It is implemented in the study and analysis of the prevention of workplace accidents as part of overall business performance [22]. It is integrated into the design phase and implemented during the building phase [24]. It is also included in studying the safety and human behavior in occupational risk prevention [25] and studying and analyzing psychosocial risks in companies [26]. Finally, it also applies during the use and maintenance of the building [27]. Not all risk-fighting techniques are covered or very complex for direct application on-site [4,5]. For all these reasons, it is necessary to identify the parameters corresponding to a construction site’s situation and encompass the techniques to reduce risk: Occupational Safety, Industrial Hygiene, Ergonomics, and Psychosociology [4,5].

Current occupational risk assessment methods adapted to construction sites’ particular characteristics use qualitative and quantitative observation and evaluation parameters [28,29,30,31,32,33,34,35]. These methodologies generally use zero-risk criteria in quantitative or qualitative assessments. This implies the assumption that the risks can be reduced to zero.

This article suggests that evaluators must correctly interpret the zero-risk concept to assess occupational risks in construction sites. So, this work aims to analyze zero-risk from a conceptual perspective to question, in risk assessment methods, the guidelines for a correct interpretation of the zero-risk concept from a technical-preventive perspective.

## 2. Concept of Non-Nullity of Risk

Risk is an abstract concept whose definition cannot be exact because it is influenced by subjective factors, including what an individual perceives as risk and the cultural and socioeconomic framework. Alayón makes a brief comment on the philosophy of risk by mentioning French psychoanalyst and philosopher Anne Dufourmantelle [36], who insisted that “the charm of risk is that it is in life,” since life itself is risky and can only be risked from life. This perspective allows a person to feel alive: “Living without risks is really not living.” Becoming aware of the wonder that risking life represents, that is, taking the risk of living, is to be ready to assume that there are more risks in life, which are unique and unrepeatable for each individual.

The individual takes a risk because he has understood that “when a danger has to be faced, there is a very strong temptation to take action, to sacrifice oneself” and, consequently, when risking, one reaches “an intensity that is a way of being reborn”, even if by that action he loses his life [37].

On the other hand, Korstanje defines risk as a context that moderates the negative effects of uncertainty on the body and its psychology, establishing that risk is a matter of likelihood. Although it considers that, in general, individuals can access all the causes that show the likelihood of avoiding danger because the nature of the individual tends to fear what is beyond our control. Risk is a concept born from the speculative and financial world that corresponds to a form of value. All risk implies a decision maker’s prior decision to avoid danger or contingency [38].

In applying these philosophies to construction sites, Rodríguez points out that risk management and administration encompasses the prediction and anticipation of events that may cause undesirable results. In sum, risk is an abstract concept that is quite complicated to define and, in many cases, impossible to measure accurately. The degree of risk can be very different based on the nature and characteristics of each project. The risk would be defined as the contingency, likelihood, proximity of danger or damage, and a negative event that could involve physical damage and economic loss. International practice and doctrine have tended to transfer risks to a third party.

Consequently, a construction company often bears the vast majority of a project’s risks without considering the capacity it may have to evaluate and lessen these risks [39]. Historians have determined that from the Age of Enlightenment (or Age of Reason—17th to 18th centuries) to our time (at the beginning of the twenty-first century), these approaches were decisive, achieving financial and personal benefits in companies. However, despite the controversies generated by the preventive environment in project management, it is necessary to give more importance to the responsible person for decision-making on occupational risk prevention. The importance is because this person is continually considering any arguments about constructive systems and risk prevention that destabilize the economic-personal equilibrium [40].

## 3. Review of Occupational Risk Assessment Methodologies

The length of the bibliography illustrates the difficulty of establishing joint criteria for risk assessment in construction works [1,2,3,4,5,12,13] due to the construction sector’s particular characteristics [14,15,16]. The common practice in evaluating occupational risks in construction works is technical observation [10]. This approach requires qualitative linguistic [41] and quantitative criteria with numerical values [42].

This section offers a critical analysis of current occupational risk assessment methods based on the particular characteristics of construction sites. These methods incorporate different risk-fighting techniques together. The methodologies reveal an evolution in the areas of analysis and evaluation. In addition, they incorporate new parameters based on observation and measurement criteria with the use of the zero-risk concept and its interpretation [24,28].

### 3.1. Construction Sites Risk Assessment Tool

Forteza et al., developed the Construction Sites Risk Assessment Tool (CONSRAT) methodology [29]. They noted that there is little existing information about risk assessment methodologies adapted to building works. Generally, specific analysis methodologies involve a detailed study of techniques to combat risk. These methodologies are not sufficient to observe the risk conditions in building works accurately because of the characteristics of this type of work [28]. This method obtains generalized information from the work’s characteristics, the promotor, the contractor, documentation, environmental conditions, the specific risks of the work, protections, auxiliary systems, and machinery. This methodology proposes a simple tool with 11 risk variables that characterize the companies involved and relates them to the analyzed variables. It collects information about the work environment, how it is structured, the physical development, the agents, and the type of work to identify and assess risks, barriers, and auxiliary systems. In the structure of the methodology, each of the observation items is provided with an identifying variable that is quantified based on the experience of the evaluator. The quantification values range from 0 to 1, applying dichotomous criteria (e.g., yes/no, correct/incorrect, 1/0, presence/absence). The criteria for evaluating the global risk level are categorized based on the final result. For values from 0 to 0.33, the risk is considered good; for values from 0.33 to 0.66, it is acceptable; and for values from 0.66 to 1, the risk is unacceptable.

The criterion that indicates the absence of risk or very low-risk levels corresponds to values between 0 and 0.33. However, considering the characteristics of construction works, it is anomalous that the average value of the evaluation could be exactly “0” or close to “0”. During the evaluation process, the level of controlled or non-existent risk corresponds to the value “0”. The interpretation that best fits this value is that there is no risk or an absence of risk.

### 3.2. Qualitative Risk Assessment Method

Pinto developed the Qualitative Risk Assessment Method (QRAM) as another risk assessment methodology adapted to construction sites [30]. It is a versatile tool for use on construction sites that uses and prioritizes linguistic variables that focus on the best way to determine risk factors to represent a more objective and reliable risk assessment. This risk assessment method is based on an Event Tree. It has four dimensions: adequacy of the safety climate (SC), severity factors or consequences when a work accident occurs (S), factors of possibility or likelihood of occurrence of an accident at work (AP), and effectiveness of the safety barriers or safety systems (SB). It is applied to the following characteristic risks: falls, electrical contacts, blows by moving vehicles, injuries due to falling objects, collapses in excavation, blows from rolling or slipping, contact with moving machines and tools, losing buoyancy in water, fire, and explosions in confined spaces. These risks represent 98% of the accidents that occur on construction sites [43].

Although it is a global method that encompasses four techniques to evaluate risk, it lacks simplicity for direct use on-site in the decision-making hierarchy because it requires a very extensive sampling procedure. However, the results in applying the different mathematical expressions are framed from the value 0 to the value 1, both inclusive. Contexts evaluated as “strongly adequate”, and “excellent” for risk control can have values ranging from 0 to 0.06, where 0 is an included value. One could reasonably conclude that for a construction site where the assessed value is “0”, this method determines there is no risk in the safety climate, no risk of an accident occurring, and even no possibility of its occurrence.

### 3.3. Method for Multiple Attribute Decision Synthesis + Sensitivity Analysis

Simanaviciene et al., developed the SyMAD-3 + SA occupational risk assessment methodology [31] and determined that there is currently no approach that ensures occupational safety comprehensively. Many scientists create new methods related to health and safety, but many of them analyze safety aspects in the construction field separately. However, the implementation of these evaluations can cause doubts about the decisions made in construction always associated with uncertainty. The decision-making process is defined by selecting different alternatives based on criteria to achieve one or more objectives [44]. Decision-making begins by identifying the problem and ends by selecting and implementing an alternative. This methodology follows a sensitivity analysis algorithm within a decision flow. For the effectiveness of the flow development, it is necessary to establish selection criteria “1”, “or”, “and”, and “0”. When the flow value is zero, a new decision matrix must be generated. This structure indicates that to make a decision means that there is no risk, so this decision must be evaluated as correct, taking the value “0” in the flow diagram.

### 3.4. Work & Organization Network

In the Work & Organization Network (WONT) methodology, developed by Salanova et al. [32], not only are psychosocial risks and dangers detected, but the methodology also recommends organizational and individual strategies for the prevention of work stress to optimize the health and well-being of the organization. Its objective is to achieve “healthier” organizations, considering the four fundamental dimensions of environmental factors, physical health, mental health, and social health [45]. The methodology is based on Resources, Emotions, and Labor Demands (RED) questionnaires designed by Kasarek and Theorell [46,47], with the identification of psychosocial risk factors (company, workgroup, department, etc.), the identification of potential risk groups (older workers, temporary workers, low-skilled workers, immigrants, etc.) and the identification of people at risk of developing stress-related problems (technostress, burnout, work addiction, mobbing, etc.). The model establishes a relationship among demand, control, and social support as psychological demands concerning the psychological needs that work implies. Because the methodology refers to how much work is done (amount or volume of work, time pressure, level of attention, unforeseen interruptions), it is not limited to intellectual work but can apply to any task type. Being overconfident is one of the behavioral parameters that most influences and generates occupational risk in construction sites [48].

The results cover an abstract range (from lowest to highest) within the context of stress and the possibility of illness. An evaluation of positive psychosocial well-being (engagement, flow, satisfaction) is identified with a situation of little risk, low risk, or no risk. This would imply an effective learning motivation response to develop new behavioral patterns, removing the risk of psychological stress and physical illness. Of course, when faced with a situation evaluated with positive or low-risk characteristics, a worker can respond with overconfident behaviors [49,50].

### 3.5. Integrated Sustainable Index for Health & Safety Engineering

Reyes et al. [33] analyzed health and safety criteria to determine the sustainable value in construction works. Health and safety concepts offer the possibility of minimizing rates of unsafe actions and reducing project costs through the four phases of a building’s life cycle: design, construction, useful life, and reintegration. This methodology asserts that planners must incorporate issues related to the economy and social terms to promote sustainable development. This methodology is based on subjective criteria based on decision-making regarding global factors such as the project, the safety study, the work budget, the health and safety budget, and the safety coordinator’s work. The evaluation considerations of this methodology are abstract. However, it is quantified on a 0–100 scale by the evaluation criteria described in health and safety documents. The evaluation is quantified from 0 to 20 when minimum standards of the health and safety Spanish legislation are fulfilled; from 20 to 50 when instructions for workers and their tasks are covered; and from 50 to 100 when the evaluation identifies work procedures, execution specifications, memory, and correctly referenced plans. This methodology considers sustainable approaches and covers pervasive temporal conditions, so the items are global.

In interpreting the results of this procedure, the null risk concept is reflected in the valuation closest to 100. This implies that the project documentation, plans, health and safety documents, work documents, and work procedures are correctly referenced. It does not particularise an evaluation of each risk during the execution phase of the building. Therefore, this assessment examines the managerial hierarchy: developer, contractor, designer, project manager, and health and safety coordinator. It is important to note that many investigations determine that one of the factors affecting workplace accidents is a lack of proper project documentation [15,24,51].

### 3.6. Integrated Methodology for the Assessment of Professional Risk

Oliveira [34] proposes an integrated global risk assessment methodology. She justifies her research base in that accidents, incidents, and diseases are due to human error, machines or materials, sociology, and health. To this end, the methodology proposes that risks can be described, characterized, and classified according to their causes, the analysis of their consequences, and the possibility of configuring a coherent, reproducible, and comparable methodological evaluation system. This methodology builds on the classical methodology of the tree of causes and effects, “Hazards and Effects of Management Process (HEMP)” [52,53], expanding it with four types of event trees. The procedure is called “Management Process of Causes, Decisions, Effects, and Faults (CDEF)”, and the data treatment is probabilistic. As a fundamental pattern, the study is based on the three basic concepts in risk, in the following logical expression: the event implies the occurrence. The occurrence implies the consequence. In the risk concept, there are two barriers to the objective: general (physical, human, organizational, and psychosocial) and specific (prevention and protection).

This methodology does not use null risk evaluation criterion or a value of “0”, but for the monitoring and implementation of the four-event trees, it is necessary to establish the correspondence of the value “1”, “or”, “and”, and “0” in the flow diagram. The great difficulty in obtaining a null result means that this method has a range of possibilities adapted to construction works’ complexity. In this case, the interpretation of small risk, low risk, or no risk does not fit; what happens is a very low likelihood of an adverse event occurring, with a value of “0”. In the statistical formula for applying the method, values range from 0 to 1, without both being included.

### 3.7. Method for the Evaluation of Occupational Risk in Construction Works of Large Viaducts

Finally, the research carried out by Claudino [35] proposes a method for the evaluation of occupational risks in the construction of large viaducts. The new model’s global design consists of a system for analyzing a work environment, obtaining a series of results that indicate, in a structured way, its level of safety against the risks of work accidents. The method consists of three parts. The first is a protocol of requirements for the object of analysis, “Protocol for bridge and viaduct construction works—OC/PV”, the second is the “Data analysis system for occupational risk assessment—ERL”, and the third is the “Risk control procedure—NC”. The methodology establishes two fundamental risk factors based on environmental risk (physical, chemical, and biological) and safety risk (accidents, organization, ergonomic, and psychosocial). The method provides the necessary information to know whether the work situation is not compliant (or compliant) in its conformity to the regulations and provides a percentage value based on a sum of compliant or non-compliant aspects. The percentage does not reveal the degree of risk, so non-conforming requirements need a more detailed analysis. The method identifies where the dangerous conditions are and the operators associated with those dangers. It is a method that requires work knowledge, an understanding of health and safety considerations, and computer science methods. The procedure quantifies, with values from 0 to 1, each of the 124 questions globally. It includes evaluations of the management, work organization, working conditions, fall protection, tools, machinery, machinery for work at height, scaffolding, electrical installations, welding, personal protective equipment, structures, and construction systems.

According to the methodology questionnaire, a range of values between 0.95 and 1 indicates little risk, low risk, or no risk because the project has met the legal requirements. The methodology defines such a score as a safety index of a moderate nature. In this methodology, the concept of zero-risk or no risk is very appropriate. Although the interpretation of the term “moderate” may be very subjective, it does not imply absolute safety or zero-risk.

## 4. State-of-the-Art Regarding Zero-Risk

Risk is a difficult concept to clarify and evaluate. Many authors define, with very similar criteria, the concept of risk as a measure of the magnitude of damage concerning a situation that denotes danger [54]; as the likelihood that a worker will be injured or have an adverse effect on health after being exposed to a hazard [55]; or, as the relationship between the parameters of risk, likelihood, and consequences [56,57,58]. However, it is complex to define the magnitude of risk, both qualitatively and quantitatively [59,60]. In any method of occupational risk assessment, two limits are characterized linguistically by the conditions of “maximum risk” and “minimum risk” [54]. As mentioned above, in the different evaluation methodologies, evaluators employ linguistic expressions such as little-risk, zero-risk, no-risk, trivial-risk, or similar statements for both criteria. Quantitatively, “minimum risk” is usually identified with the value “0” and “maximum risk” with values other than “0”; generally, “1” or “2” [28,29,30,31,32,33,34,35]. When a situation suggests “maximum risk” or a tendency toward it, a worker usually expresses a desire to avoid such a situation [61]. However, the difficulty comes in defining a situation with zero-risk. In what context does a worker feel safe?

Section 3 presented different risk assessment methodologies and their adaptation to construction projects and their assessment values. This section describes the compilation of a bibliography using the search terms zero-risk, health and safety, and construction. It analyses this bibliography regarding the interpretation provided by each of the authors on the meaning of “Null Risk”, including similar terms and even antonyms, how they assess such risk, and the worker behavior they expect as responses.

### 4.1. The Use of Zero-Risk in the Assessment of Occupational Risk

Mora [54] discusses the general techniques of analysis, evaluation, and control of occupational risks. Mora analyses the risk and its interpretation with the concepts of dangerousness (likelihood of occurrence of a hazard), vulnerability (likelihood of damage when a hazard has occurred), and risk (extent of damage against a dangerous situation). In the analysis of risks, Mora establishes a possible scale of values for its better interpretation from zero-risk to maximum risk. The latter is at the limit of the danger concept. The study then presents different situations of acceptable risk on the scale [54].

In Rawlinson and Farrell’s [62] research on zero-risk tolerance among artisans, they mention the need for certain construction workers to consent to assume a specific risk. They found a high tolerance for risk along with the need for such tolerance given the nature of the work, but no desire to engage in reckless behavior. Everyone takes risks, albeit of different natures, and in part the risk is what attracts some people to some work. In an industry considered macho and dangerous, studies have shown that many people who are attracted to construction work display these characteristics at a much higher rate than those who work at selling cars, for example [63]. Construction workers like to take risks [64]. They want to climb higher and tunnel deeper than most people. The general population often views construction as risky [65]. However, construction workers often do not consider their work hazardous even when the recognized safe working limits for the task are stretched or broken.

In research on engineering considerations in risk tolerance, Joughin [66] performed a critical analysis when making judgments about tolerable levels of risk in the Mining and Metallurgy industry and in all spheres of life. Joughin explained that each action gives rise to associated risks, and risk is part of life and the economy. There is a strong tendency to emphasize risk at work, but it exists in every act of life at work or away from it. Often, people who are not working face more significant risks than people who are working. Simply by being alive, any individual is exposed to an extremely diverse range of risks. For Joughin, a zero-risk context is impossible because “there is no natural state of zero-risk. Because zero-risk is physically impossible, nobody can design a zero-risk project. While the strategic objective of achieving zero-risk is laudable, it does not provide any practical guidance to engineers on what the acceptable limits of risk should be. This fact causes a significant and real problem in project design and management. Without meaningful guidelines, engineers, managers, and authorities often disagree. Therefore, establishing criteria for tolerable risk levels and unacceptable risk levels has become essential to properly design and manage engineering systems. However, perceptions of unreasonable risk fall into two distinct categories. In one category, there are perceptions of unreasonably low levels of risk. In the other category, people are willing to take unreasonably high risks.

Zwetsloot et al. [67] conducted research on strategies companies established based on the goal of zero accidents. According to further research, the authors analyzed the zero-accident goal following the Safety Science guidelines in 2013. The latest research on risk assessment presents approaches from the perspective that all accidents are preventable rather than the abstract concept of “zero accidents” as a goal. In this case, the prevention approach is focused on zero accidents or a goal of zero accidents. This program implies the development of a preventive culture. In particular, a preventive culture “driven by ZAV (Zero Accident Vision)” emerges from shared values and practices such as vigilance and shared awareness, questioning attitudes, and a willingness to make sense of safety procedures and devices. A safety culture that promotes greater engagement and empowerment is not compatible with traditional bureaucratic controls. This approach does not exclude all controls; ZAV is a combination of shared knowledge about a dynamic environment and a willingness to anticipate and keep the system secure. Therefore, methods and resources, such as leading indicators and flexible communication systems, are necessary to support an efficient exchange of information between people.

The concepts of safety and danger are related to workers’ perception of their environment, attitudes, and personalities [68]. Pourmazaherian et al. [69] described the role of five significant personality factors in construction accidents. Among the psychological factors that can affect the attitude of workers are neuroticism, with a very direct relationship to workplace accidents; extraversion, whose relationship with accidents is moderate; frankness, which implies that individuals tend to be imaginative, curious, and unconventional, which may imply that they are more prone to breaking the rules, experimenting, and improvising, whose relation to safety may not be adequate; kindness, whose lower emotional excitement and ability to cooperate with others is related to the low incidence of accidents; and conscientiousness, whose individuals have a high level of self-control, which is positively related to safety performance.

As a fundamental condition in risk assessment, it is essential to highlight the need to observe workers’ behavior during their work assignments. Therefore, the evaluator’s categorization must be performed with adequate empathy to evaluate the necessary approximation in the null-risk context.

### 4.2. Methodology of the Level of Preventive Action

The new risk assessment method establishes the prevention level, deviating from the initial approach, in the Occupational Health and Safety Plan. This method determines the level of preventive action (Lpac) that needs to be incorporated into the development of a project to improve the design conditions in the initial and documentary environment, the work conditions in the construction environment, and relationships and behaviors in the social environment [5,10,70]. It is a method adapted to the “special” complexity of construction projects. It encompasses an evaluation of four of the techniques to fight risk: Occupational Safety, Industrial Hygiene, Ergonomics, and Psychosociology, assigning the same degree of importance to each of the methods. The parameters that define the Lpac are:

- Probability (P) and Consequences (C) in the absolute or initial environment from the basic parameters of risk.
- Relative Risk (Rr) and Border Risk (Rb) in the documentary environment are based on the building’s physical and geometric parameters.
- Degree of Exposure (E) and Economic Capacity (Ec) in the construction environment, based on the construction means’ parameters and exposure of the workers to the risk.
- Participatory interest (Pi) and Level of Satisfaction (Ls) in the social environment based on the emotional state and participation parameters.

The mathematical expression (1) that defines the Level of Preventive Action (Lpac) is characterized by the Absolute Risk (Rab), which is expressed by the direct relationship between the likelihood and the consequences; on which a correction coefficient, called the Evaluation of Preventive Action (Epac), is applied that depends on six variables linked to the documentary, constructive, and social environments. The list of parameters that express the Level of Preventive Action based on Absolute Risk and the Environment Assessment parameters can be described as follows:

L_pac_ = (R_ab_)·(E_pac_) = (P·C) · ((R_r_·R_b_·E)/(E_c_·P_i_·L_s_)) (1)

**Figure 2:**
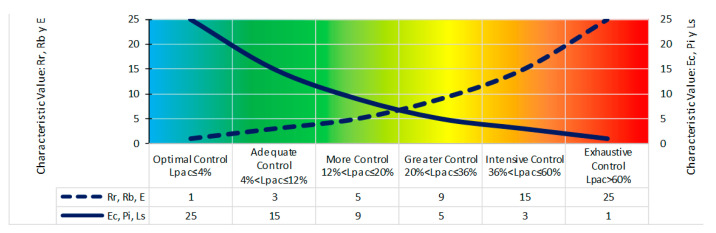
Characteristic Values and Control Bases of the Level of Preventive Action.

Each of the Level of Preventive Action formula parameters is quantified as one of the following integer values: 1, 3, 5, 9, 15, and 25 (Figure 2). This quantification is called the characteristic value of the Level of Preventive Action, a new concept of evaluation and observation associated with the real characteristics presented by the building unit in execution on which it is going to be evaluated. It is based on the work unit’s complexity criteria, the work unit’s location, the degree of worker exposure to risk, the organizational procedure of the work environment’s general protection systems, participatory interest in prevention, and perception of the congruence of risk in the work environment. This quantification determines a base value, a priori, of the risk associated with the work unit’s current conditions. For the Relative Risk, Border Risk, and Degree of Exposure parameters, the characteristic value that identifies the minimum risk is 1 [5,10]. For the Economic Capacity, Participative Interest, and Level of Satisfaction parameters, the characteristic value that identifies the minimum risk is 25 [5,10].

**Figure 3:**
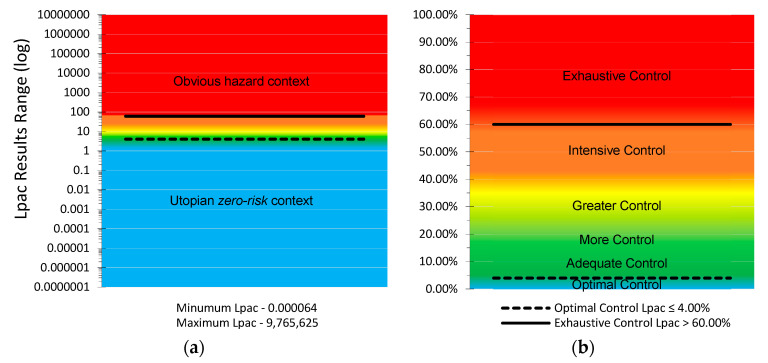
Values Range of the Level of Preventive Action. (**a**) Graph with scale logarithmic on the ordinate axis. (**b**) Graph more intuitive, where the results are shown in percentages and are identified by colors the preventive action control bases of the method.

In both cases, the minimum value to evaluate, 1, is in the very nature of its mathematical expression. The result of the method must be a non-nullity. The Level of Preventive Action results are expressed without units and cover the range of values [0.000064 to 9,765,625.00] (Figure 3). In Figure 3a, the scale shown on the ordinate axis is logarithmic. However, the results are shown in percentages (Figure 3b). In the preventive action control bases of the method, different control criteria are established for the result, identified by colors:

- Lpac ≤ 4%, optimal control of preventive action (blue).
- 4% < Lpac ≤ 12%, adequate control of preventive action (green).
- 12% < Lpac ≤ 20%, more control of preventive action (light green).
- 20% < Lpac ≤ 36%, greater control of preventive action (yellow).
- 36% < Lpac ≤ 60%, intensive control of preventive action (orange).
- Lpac > 60%, exhaustive control of preventive action (red).

This methodology establishes that all the Level of Preventive Action results equal to or less than 4.00% corresponds to the preventive action’s optimal control situation. For an optimal control preventive action, the work context has been evaluated with controlled risk or minimal risk. This result indicates that preventive action is optimal, interpreted in a context of preventive action or a utopian goal of zero accidents [71], and does not require additional preventive measures [72]. In contrast, all the results that exceed the value of 60.00% correspond to a work context that requires exhaustive control actions. They are clearly dangerous situations [73].

When the qualitative parameters of measurement and observation indicate the non-existence of risk (or optimal control of preventive action), the agents involved in the construction project (workers, technicians, contractors, promoters, designers, and construction managers) should be able to interpret that the risk is zero or does not exist. This result generates no need for prevention or protection in the corresponding work phases [54]. It can even lead to unexpected behaviors [62,69] and overconfidence in workers [32]. This is the difficulty in observing and defining whether a situation is dangerous. Between the two conditions (danger and safety), a small region of different risks on the spectrum implies the need for various preventive actions. If the appropriate preventive measures can be applied with trained risk observation and empathy with the worker, the situation will be modified towards optimal preventive action controls, which achieves a trend towards zero-risk.

## 5. Case Study Methodological Approach

The Level of Preventive Action method has been implemented on current construction projects, establishing evaluation criteria on each of the new formula parameters adapted to the construction process. Initially, the characteristic values were calculated from technical observation and a psychosocial survey on site. Researchers then interpreted the incidence degree of each characteristic value on each risk that was evaluated. Finally, they calculated the Evaluation of Preventive Action (Epac) or environment parameter (which corresponds to the six correction parameters: Rr, Rb, E, Ec, Pi, and Ls) and, subsequently, related to Absolute Risk (Rab), calculated the Level of Preventive Action (Lpac), expressing the value without units [5,10,70].

**Figure 4:**
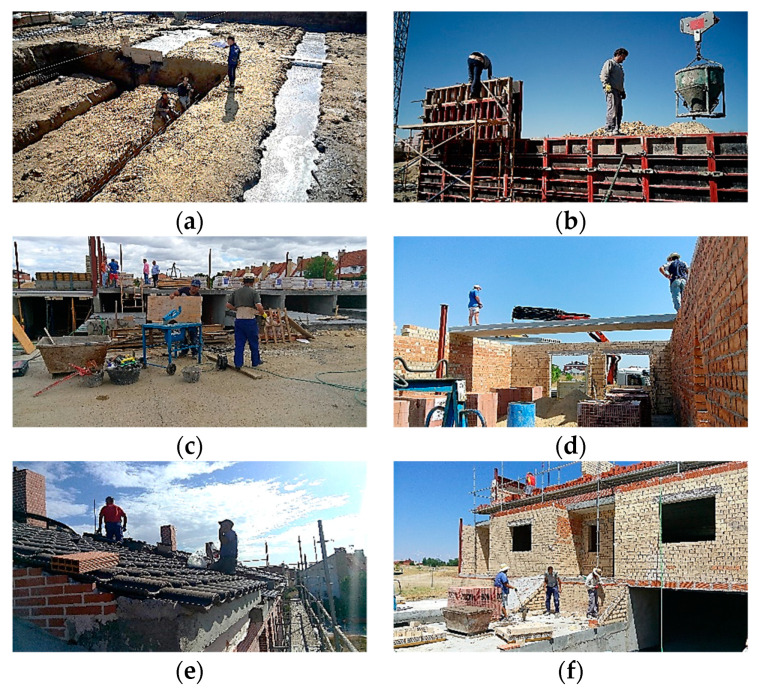
Construction systems during the different execution phases: (**a**) foundation; (**b**) reinforced concrete walls; (**c**) urbanization; (**d**) precast slabs; (**e**) roofs; (**f**) facades.

Researchers collected data from a real construction project in the province of Madrid (Spain). The project involved the construction of a building with six terraced houses on three levels, a ground floor for a garage and storage room, and two floors above ground for housing. The total constructed area of the building was 1528.26 m^2^. Researchers collected data weekly over 34 working weeks from 17 June 2016 to 27 April 2017 [10,70]. Figure 4 shows some photographs of the development of the work. The health and safety context in the different construction systems was very delicate, some with obvious high-risk situations.

Researchers collected technical data by direct observation and psychosocial data through a survey on site. The questionnaire asked workers and construction agents (promoter, designer, builder, project manager, and health and safety coordinator) about their perceptions of different risk prevention contexts [10]. The questions cover the social environment (personal perception) and the psychological environment (risk perception) (Table 1). The same questionnaires were answered weekly by the same workers during the 34 weeks of data collection [10].

Data collection is divided into two groups of workers. The first group of workers composed with superior technical training (designer, Construction Director, Project Manager, etc.), managers of the construction company, and managers of the promoter company. The second group of workers comprises those whose training is proper to the trades they perform in the construction process (Table 2). According to the inspection, the different professions that intervened during the construction and participated in the survey were identified.

## 6. Results

**Figure 5:**
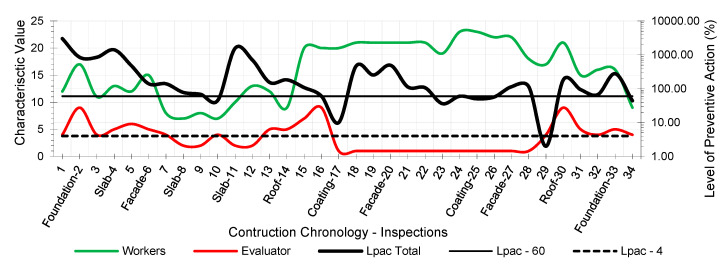
Perception of individual participation.

**Figure 6:**
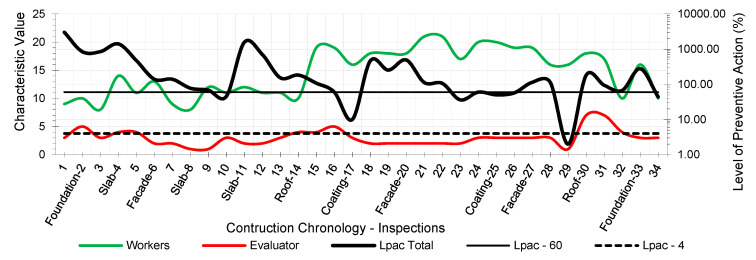
Perception of group participation.

**Figure 7:**
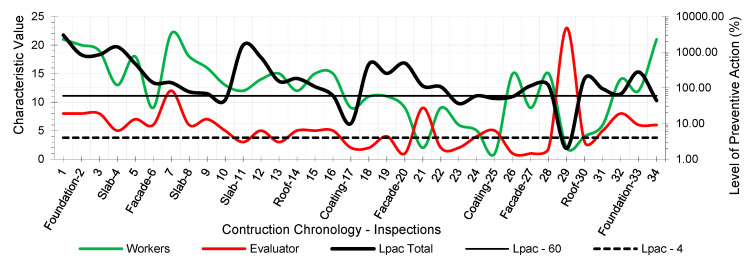
Perception of the Controlled Risk of the Work.

**Figure 8:**
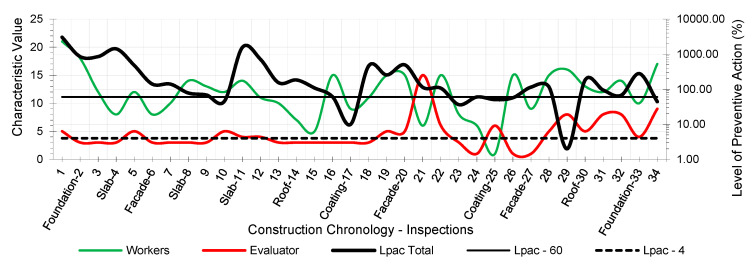
Perception of the Controlled Risk of the environment.

**Figure 9:**
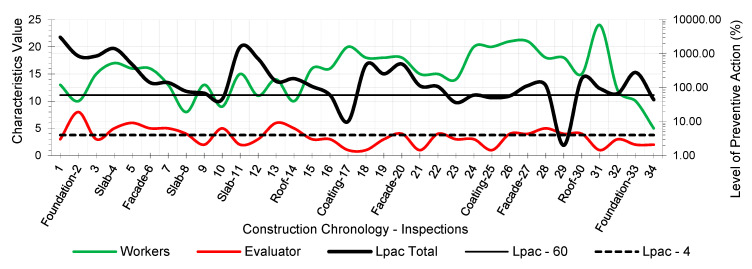
Perception of individual protection.

**Figure 10:**
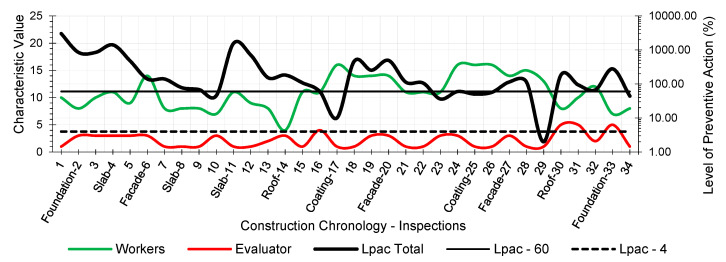
Perception of collective protection.

The results provided the workers’ risk perception concerning the necessary preventive control action to reduce the risk context to the minimum ideal. Figure 5, Figure 6, Figure 7, Figure 8, Figure 9 and Figure 10 show the results of the level of preventive action (black) and the perception of risk by the workers and the evaluator (green and red, respectively). The horizontal axis shows the correlative numbers concerning the inspection days. Likewise, the construction chronology indicates the construction systems, whose general result indicates that exhaustive preventive action control is required.

The left vertical axis represents the characteristic value of risk perception by the workers and the evaluator. The perception values from 1 for a context of danger to 25 for a zero-risk context. The results of the total Level of Preventive Action are shown on the right vertical axis. This result is the average of each of the partial products of the Level of Preventive Action regarding Occupational Safety, Industrial Hygiene, Ergonomics, and Psychosociology. The graphs show the limits for Levels of Preventive Action higher than 60.00% (which implies exhaustive control of preventive action or obvious hazard context) and below 4.00% (which means optimal preventive action control or utopian zero-risk context). The preventive measure level results show that the construction work’s prevailing perception was of very little health and safety. Most of the results are above 60.00%. Only in inspection 29 was an optimal control situation recorded.

The graphs show that workers’ perception of risk regarding health and safety differs from the evaluator’s perception. In the graphic of perception regarding individual and group participation (Figure 5 and Figure 6), from inspections 17 to 28, the workers identify that the situation is low risk even with zero-risk assessments; however, the evaluator identified the risk as maximum. Given the responses of the workers and the evaluator, the method establishes a new evaluation parameter based on the relationship of Environment Congruence responses [53]. Both are considered correct, but the method gives more weight to the evaluator’s response because it is considered to be more objective but still considers the workers’ responses. These circumstances are characteristic when workers acquire high levels of overconfidence [32], believing that they can decide to risk more by controlling the situation [38] and that the activity can be carried out safely by consciously assuming the risk [39]. The activity’s repetitiveness and routine give workers a very low perception of risk, sometimes zero, even in obvious risk situations [53], contrary to the evaluator’s objectivity. The following serve as examples:

- Site inspections 7 and 8 (slab construction). The level of preventive action was greater than 60.00% and required exhaustive control of preventive action. In the perception of individual and group participation (Figure 5 and Figure 6) and the perception of individual and collective protection (Figure 9 and Figure 10), the workers perceived it as being at significant risk (values close to 5). In the perception of controlled risk in the work environment (Figure 8), the workers perceived it as high risk. However, in the work unit’s perception of risk (Figure 7), the workers perceived it without risk, reaching a value close to 25 (*zero-risk*). On the contrary, the evaluator perceived an obvious risk context with values close to 1.
- Site inspection 17 (coating construction). The result of the Level of Preventive Action was close to 10.00%, so an adequate control preventive action was required. For the perception of participation and individual and collective protections (Figure 5, Figure 6, Figure 9 and Figure 10), the workers perceived the risk with values within the zero-risk range (values 15 and 20). However, in the risk perception of the work and the environment (Figure 7 and Figure 8), the worker perceived the risk as moderate (value 9). On the contrary, the evaluator perceived maximum risk. Economic Capacity and Participative Interest parameters correct the result of the Level of Preventive Action.
- Site inspection 29 (coating construction). The result of the Level of Preventive Action was close to 2.00%, so the preventive action had optimal control. For the perception of participation, individual and collective protection, and protection of the environment (Figure 5, Figure 6, Figure 8, Figure 9 and Figure 10), the workers perceived the risk with values close to zero-risk (value 20). However, in the perception of the work’s-controlled risk (Figure 7), the workers perceived the risk as very high (value 3). However, the evaluator perceived the existing preventive context concerning the work’s-controlled risk as close to zero.

**Figure 11:**
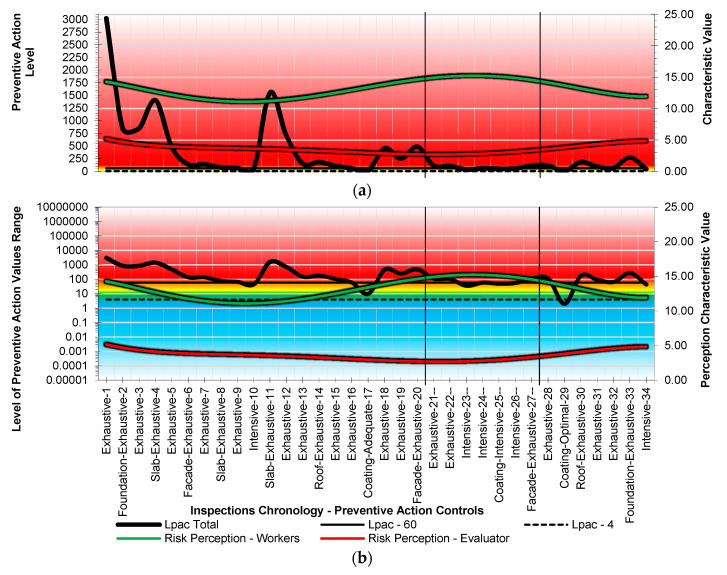
Spectrum of results. Level of Preventive Action and Risk Perception. (**a**) Results with a natural scale. (**b**) Results with a logarithmic scale.

According to the spectrum of results of the Level of Preventive Action (Figure 11), exhaustive control of preventive action was necessary for 26 of the 34 inspections (76.47%); intensive control in 6 inspections (17.65%); adequate control in 1 inspection (2.94%); and optimal control of preventive action in 1 inspection (2.94%). The dangerous risk context was obvious during nearly the entire construction process, or 94.12% of the inspections. A single low-risk or zero-risk situation could be identified. The two graphs in Figure 11a,b show the same results but with a different scale on the left vertical axis. The upper graph shows the results with a natural scale, and the lower graph shows the results with a logarithmic scale. The continuous horizontal line corresponds to the value of 60, from which exhaustive control of preventive action is required (obvious danger zone-red). The method is very sensitive to assess and detect hazards. To correct dangerous situations, preventive actions in constructive prevention systems (Ec), effective participation in prevention (Pi), and good levels of worker satisfaction (Ls) are necessary.

However, there were no accidents during building construction. No worker was seriously injured, which could be interpreted as contrary to the results obtained. Although there were many risks, workers maintained a constant perception of safety and, on many occasions, close to zero-risk (oscillating in the range of values between 10 and 15 on the right vertical axis). Regarding risk perception, the evaluator identified situations of high risk throughout the construction process (oscillating in the range of values between 1 and 5 on the right vertical axis). During inspections 21 to 27, workers perceived values close to zero-risk in the preventive environment, while the evaluator perceived maximum levels of danger, separating both trends in the graph.

## 7. Discussions

It is important to remember the particular characteristics that constructive processes have in a construction site (the concurrency of execution of different systems, interference between different work trades, variability in the development and evolution of tasks, etc.). It is extremely complex to assess the risk and to be able to check the effectiveness of preventive decision-making. However, the chronological results of the work development indicate the evolution of risk perception levels. In this respect, participation and the level of satisfaction are key elements for incorporating learning factors regarding risk perception. During the construction of a building, the results of risk perception by workers and by the evaluator should tend to the same values. This situation must be achieved through information strategies that facilitate learning and developing risk perception.

By analyzing the graphs regarding the different outcomes between workers and the evaluator, it can be determined that the perception of risk is very different from the six levels raised in the survey:

- Concerning the perception of individual and group participation in prevention (Figure 5 and Figure 6), two very different situations can be observed in work. From visit 1 to visit 16, workers and the evaluator identified similarly obvious risks during foundation, structure, facade, and roofing works. However, from visit 17 to 28, there was no risk for workers during the work of partitions, installations, coatings, flooring, carpentry, and paints. This lead to a false sense of security and behaving as if there was no risk. On the other hand, the evaluator identified alarming risk situations such as lack of railings on stairs, uninstalled windows, the formation of unstable steps, slippery floors, excess moisture, accumulation of debris, etc.
- Regarding the perception of the controlled risk of the building unit and the environment (Figure 7 and Figure 8), two other very different work situations could be observed. From visits 1 to 20, there was a clear difference between workers’ results and the evaluator; however, as of visit 20, there was a confluent trend between the results, indicating similarity between the two perceptions. During foundation, structure, facades, and roofing work, workers perceived that they had a fairly controlled risk. However, during the tasks of partitions, installations, coatings, flooring, carpentry, and paints, workers perceived the risk as under-controlled.
- With regard to the perception of individual protections (Figure 9), workers perceived that their personal protections were very good throughout the work. However, the evaluator detected a significant lack of personal protections in workers.
- Concerning the perception of collective protections (Figure 10), two situations similar to the above were detected. Until visit 14, during the construction of foundation, structure, facades, and roofs, workers perceived that collective protection was lacking. Since visit 15, during the work of partitions, installations, coatings, flooring, carpentry, and paintings, workers perceived that there were sufficient collective protections. However, the evaluator detected a great lack of collective protections in the construction.

It is important to note that, in the case study, the evaluator perceives situations and contexts of construction with obvious danger. However, workers perceive the same situations in a utopian zero-risk context. This difference shows, in a very obvious way, that the risk perception is very different from the worker’s point of view and the evaluator’s point of view. Interests and needs mark a clear opposition between the two. The Preventive Action Level method determines that the value of risk perception is the mean between the two values, with a correction of the value based on Preventive Congruence, giving more weight to evaluator perceptions. In this way, the perception of risk is assessed by the observation of the evaluator, but the worker’s perception is added. This provides a more balanced assessment criterion in the Satisfaction Level parameter. The utopian context of zero-risk will be very difficult to identify and evaluate with this methodology, approaching the generally accepted reality that zero-risk does not exist.

As might be expected in practice, the level of preventive action is quite different for the workers and evaluators (Figure 5, Figure 6, Figure 7, Figure 8, Figure 9 and Figure 10). This could mean that the level of security on the building site understudy was not in line with the applicable regulations, and it could be possible that it happened to save money. This would be the case if other risk assessment methodologies were used. In the formula applied in the Method of the Level of Preventive Action (1), used in the practical case, the denominator is the one that corrects the situation. In this denominator, there are three parameters: economic capacity (Ec); participatory interest (Pi), which is no longer money; and the level of satisfaction (Ls), which is not money either. These last two parameters are not additional economic costs but psychosocial aspects which measure the level of participation of all workers and their level of satisfaction. These three denominator factors each have a maximum value of 25, regardless of the other two factors. The economic capacity can have the maximum investment and be 25, but if the other two psychosocial factors are low, the risk is not corrected. With this reasoning, the conclusion is reached that participation in risk prevention by workers is essential. The interests of the different types of workers (technicians, including the contractor who is a stakeholder, and workers) are very different and directly influence psychosocial factors.

From a statistical point of view, analyzing the Standard Deviation of the results would lead to locating opposite critical points, locating a possible risk of accident, and finding a positive influence on prevention. For this reason, we understand that none of the responses obtained can be ruled out since, in prevention, we would waste this important source of data. One of the novelties of this method compared to those studied is that it considers the perception of risk of the different types of workers, it being one of the factors that correct the risk assessment of the Level of Preventive Action. Since the level of satisfaction is located in the denominator, the risk is corrected. The greater the value of the denominator, the greater is the correction of the Level of Preventive Action degree.

## 8. Conclusions

### 8.1. Conclusions of Theoretical Interpretation of Zero-Risk

The Knowledge Gap is that the assumption of zero-risk is not correct since it does not exist and, in addition, the perception of risk depends largely on the individual or group of people considered. The literature regarding the conceptual approach does analyze zero-risk. Risk assessment methodologies mention that zero-risk does not exist, but they do not develop this concept. Some of them even indicate that zero-risk does not exist, but at the same time, they contradict each other, using the absolute 0 value in risk evaluation.

Based on the abstract concept that encompasses risk both in its minimum and maximum expressions, the most appropriate way to assess that there is little risk is from a preventive perspective of safety and well-being. The evaluator must observe the prevention environment and pay particular attention to the workers’ perception, as defined by the Level of Preventive Action methodology, regarding the amount of preventive action required for the construction process (absolute, documentary, constructive, and social environments). The Level of Preventive Action result is focused on a preventive understanding of optimal control, as interpreted in a context or goal of zero accidents. The result of the Level of Preventive Action does not imply the absence of risk but rather determines the amount of preventive action required in a more positive and constructive interpretation.

There is a very narrow margin between a dangerous context and a safe context. Faced with a risky situation, it is essential to modify it with all intervention areas (Occupational Safety, Industrial Hygiene, Ergonomics, and Psychosociology). Maintaining these areas of intervention helps minimize risk. However, conditions of safe context, optimal control, or zero-risk can cause misbehaviors in workers who become overconfident and have a very particular perception of risk. Maintaining a context of optimal control of preventive action or zero-risk requires insights from the psychosociology discipline.

Constructing a building is characterized by complexity and constant evolution of the construction techniques by the organizational and economic-temporal planning processes. Variables include the handling of products and substances; inclement weather; the physical and mental burden of the jobs; and the roles, behaviors, and relationships of the workforce in their entire hierarchy. Those responsible for occupational risk prevention must apply psychosociological strategies throughout the construction process to improve participatory interest in the prevention of risk and the level of worker satisfaction. The purpose of these actions is to identify more effectively where the zero-risk limit is and take optimal preventive actions.

### 8.2. Conclusions Regarding the Preventive Action Level Method Implementation to the Case Study

This study determines the context of risk and how the individual perceives it in his search to feel alive. Consequently, the worker assumes the likelihood condition by proximity to the hazard. In this perception, the individual chooses to avoid danger and work with the utopia of zero-risk. The effectiveness of those responsible for preventing occupational risks in construction sites lies in knowing how to interpret, with proper observation and perception, when the workers’ prevention environment can be evaluated with zero-risk context. In the Level of Preventive Action assessment method, results below 4% indicate that the preventive environment is safe.

By implementing the Level of Preventive Action method at an active construction site, this study collected risk assessment results that demonstrated that the work environment was very dangerous throughout the construction process. However, the workers had a perception of risk that was contrary to the evaluator’s perception, including even a perception *of zero-risk* in some dangerous conditions. For an adequate evaluation of occupational risks, the data were contradictory, so it was advisable to establish a criterion that gave more importance to the evaluator’s assessment. The method applies a criterion that relates the perception of risk between the worker and the Environment Congruence evaluator. This criterion favors or penalizes the Characteristic Value of the Level of Satisfaction parameter. Faced with a context of danger, when the Environment Congruence is the same (value 1), it indicates that the perception of risk is congruent between the worker and the evaluator. This situation makes the control of preventive action tend to be optimal in a dangerous environment. This result indicates that the zero-risk context has different interpretation levels, so it depends on the different observation environments: the initial environment, documentary environment, constructive environment, and social environment. When the Level of Preventive Action is optimal, it means that the risk is low in all preventive observation settings. Therefore, both the circumstance of dangerous environments and optimal preventive action results can occur simultaneously. So, this leads to understanding the evident difficulty of measuring risk as zero in construction sites.

In the parameter of the Satisfaction Level, the survey is carried out. Workers are asked about six different levels of risk perception: Perception of individual participation, Perception of group participation, Perception of the Controlled Risk of the Work, Perception of the Controlled Risk of the environment, Perception of individual protection, and Perception of collective protection. The graphs show the perception of risk regarding the responses of the workers and the evaluator, the results showing a huge difference between the two. This situation shows a very common problem in construction sites regarding overconfidence. However, it could also be proposed as a paternalistic approach regarding excess prevention. In this situation, it is important to consider the separation between the two results. The closer they are, the Preventive Congruence value will approach 1. This will indicate that the worker and the evaluator will have the same perception of risk (both in an obvious hazard context and in a utopian *zero-risk* context) for what may be appropriately proposed agreed preventive action controls.

Occupational risk assessment methodologies must incorporate risk spectrum assessment criteria, including zero-risk. Risk is a subjective and abstract concept, difficult to observe and measure. The absence of risk is even more difficult to determine. However, the charm of risk is that it is in life and is kept in mind. Every risk implies a prior decision; that is, unwanted events must be predicted and anticipated. The degree of risk depends on individual perception. The concept of zero-risk is too abstract and reckless to identify the absence of risks or dangers. Given the complexity of risk and the difficulty of its evaluation, the health and safety of construction projects must be approached from an environment of preventive culture, with a preventive attitude to safety and well-being, in a more positive and empathic interpretation. Learning to identify zero-risk can help to interpret the magnitude of risk in the preventive environment.

In reading the literature regarding zero-risk and analyzing the different risk assessment methodologies regarding zero-risk treatment, it is necessary to propose a different approach in risk assessments, particularly on construction sites. It is necessary to know the behavior of risk assessments compared to zero-risk assessment, checking that they adapt to the generally accepted reality that zero-risk does not exist. Therefore, evaluations with qualitative and quantitative non-risk approaches should be discarded.

In conclusion, this study justifies the non-nullity of the risk and the difficulty of assessing zero-risk in construction sites. Therefore, the assessment should be approached from an environment of preventive culture, tending that the perception of the risk of the worker and the evaluator is the same. This is the congruence, which allows workers to participate actively in the control of preventive action, promoting that preventive measures are properly implemented in hazardous environments.

## Figures and Tables

**Table 1:** Psychosocial Survey. Satisfaction Level Parameter.

Social Survey			Psychological Survey				
Personal Perception Questionnaire			Risk Perception Questionnaire				
What do you consider your emotional state to be?			What complexity level or difficulty do you associate with the work that you are doing?				
What would you say is your current energy level?			What is the level of danger associated with the work that you do?				
What is your level of personal satisfaction?			How high is your level of participation in your own safety?				
What is your level of job satisfaction?			How high is your level of participation in your colleagues’ safety?				
			To what level do you consider your individual protective equipment to be complete?				
			To what level do you consider the collective protection systems to be complete?				
**Responses**			**Responses**				
Workers		Characteristic Value Corresponding	Workers		Assessor		Characteristic Value Corresponding
Bored	1	**1**	Zero	1	Zero	1	**1**
Upbeat	2	**3**	Very slight	2	Very slight	2	**3**
Motivated	3	**5**	Slight	3	Slight	3	**5**
Focused	4	**25**	Notable	4	Notable	4	**9**
Challenged	5	**15**	Considerable	5	Considerable	5	**15**
Excited	6	**9**	High	6	High	6	**25**
			**Congruence Preventive analysis between the worker and the evaluator responses**				

**Table 2:** Identification of construction trades for each inspection for the survey.

Construction Workers	Weekly Inspections																																	
	1	2	3	4	5	6	7	8	9	10	11	12	13	14	15	16	17	18	19	20	21	22	23	24	25	26	27	28	29	30	31	32	33	34
	June		July					August				September			October				November			December	January				February			March			April	
**Promotor, Constructor and Technicians**																																		
Promotor Manager	X		X				X	X	X	X	X											X	X		X									
Builder Manager	X		X				X	X	X	X	X											X	X		X									
Architect	X		X				X	X	X	X	X											X	X		X									
Project Manager	X		X				X	X	X	X	X											X	X		X									
Safety Manager	X		X				X	X	X	X	X											X	X		X									
**Construction Trades**																																		
Foreman	X	X			X			X			X	X		X				X			X					X					X			
Excavator Machinist	X		X																														X	
Truck Driver	X		X																														X	
Carpenter of structure		X	X	X	X	X	X	X			X	X		X																			X	
Steel Fixer		X	X	X	X	X	X	X			X	X		X																			X	
Crane Operator											X	X		X		X	X	X	X	X	X	X											X	
Cement & Concrete Finisher		X	X	X	X	X	X	X			X	X		X													X	X	X	X	X	X		X
Bricklayer				X	X	X	X		X	X	X		X		X	X	X	X	X	X	X	X	X	X	X	X	X	X	X		X	X		X
Ironworker											X				X																			
Laborer				X	X	X	X		X	X	X		X		X	X	X	X	X	X	X	X	X	X	X	X	X	X	X		X	X		X
Flooring Installer					X																				X	X				X	X	X		X
Roofing Worker																		X	X	X	X	X								X				
Electrician																												X	X					
Plumber		X	X																	X								X	X					
Sanitation Facility		X	X																						X				X					
Plasterer																							X	X	X		X	X	X	X		X		
Carpenter and Glazer																											X	X	X	X				
Painter																											X	X	X	X				
HVAC Tech																					X					X		X	X					

## Data Availability

The data presented in this study are available on request from the corresponding author. The data are not yet publicly available due to the authors continue to use them for research purposes.
